# Noise Minimisation in Gene Expression Switches

**DOI:** 10.1371/journal.pone.0084020

**Published:** 2013-12-23

**Authors:** Diana Monteoliva, Christina B. McCarthy, Luis Diambra

**Affiliations:** 1 Laboratorio de Biología de Sistemas, Centro Regional de Estudios Genómicos, Facultad de Ciencias Exactas, Universidad Nacional de La Plata, La Plata, Argentina; 2 Laboratorio de Metagenómica de Microorganismos, Centro Regional de Estudios Genómicos, Facultad de Ciencias Exactas, Universidad Nacional de La Plata, Florencio Varela, Argentina; 3 Departamento de Informática y Tecnología, Universidad Nacional del Noroeste de la Provincia de Buenos Aires, Pergamino, Buenos Aires, Argentina; 4 Instituto de Física, Universidad Nacional de La Plata, La Plata, Argentina; Tata Institute of Fundamental Research, India

## Abstract

Gene expression is subject to stochastic variation which leads to fluctuations in the rate of protein production. Recently, a study in yeast at a genomic scale showed that, in some cases, gene expression variability alters phenotypes while, in other cases, these remain unchanged despite fluctuations in the expression of other genes. These studies suggested that noise in gene expression is a physiologically relevant trait and, to prevent harmful stochastic variation in the expression levels of some genes, it can be subject to minimisation. However, the mechanisms for noise minimisation are still unclear. In the present work, we analysed how noise expression depends on the architecture of the *cis*-regulatory system, in particular on the number of regulatory binding sites. Using analytical calculations and stochastic simulations, we found that the fluctuation level in noise expression decreased with the number of regulatory sites when regulatory transcription factors interacted with only one other bound transcription factor. In contrast, we observed that there was an optimal number of binding sites when transcription factors interacted with many bound transcription factors. This finding suggested a new mechanism for preventing large fluctuations in the expression of genes which are sensitive to the concentration of regulators.

## Introduction

Living organisms sense and respond to environmental clues in order to obtain energy resources and overcome stressful conditions. This is achieved by employing gene regulatory networks, also called gene circuits. Each circuit acts as an input-output device which is designed to be activated by a specific signal and to elicit the required response. The dose-response curve of a given genetic circuit can be described by a continuous function, also called the regulatory function, which relates to the intensity of the input signal and the magnitude of the output response. Two important aspects of the regulatory function include its sensitivity (linked to the steepness of the function) and the apparent dissociation constant (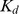, equivalent to the stimulus intensity required to obtain half the response). In gene expression, a critical feature of the output response is its inherent variability. Given the small number of molecules involved in the biochemical processing of signaling (gene transcription [1], chromatin remodeling [2], formation of transcription reinitiation complexes [2], [3] and protein translation [4]), this results in variable protein concentrations across cell populations. This phenotypic variation can affect survival [3], [5], [6], [7], [8], [9], differentiation [10], [11], [12], [13], [14], [15], [16] and also increases evolvability [17]. Thus, biochemical circuits must have evolved to maximise the overall performance of the organism. Sometimes, the evolutionary optimisation process can be constrained to obtain a robust system (i.e., insensitive to the precise values of biochemical parameters). In other cases, biochemical circuits need fine-tuned intracellular parameters and, consequently, inherent biochemical noise must be minimised.

Several studies have suggested the existence of optimisation criteria in the design of some regulatory systems [18], [19], [20], [21], [22], [23]. Of course, this requires one or more functionality criteria operating on the course of evolution. Among these, cost-benefit (the trade-off between the metabolic costs of protein synthesis and the benefits of protein function [24], [25]), maximisation of information transmission [21], [20], [26] and minimisation of biochemical noise [20], [27], [28], have been mentioned as functionality criteria for an optimal design.

In particular, the latter has been addressed for multistage signaling cascades in which several genes are involved [29]. Nonetheless, the existence of such a criterion operating as a design principle at single-gene level has not yet been explored. On the other hand, there have been recent advances in identifying and characterising a variety of mechanisms involved in the regulation of gene expression [30], [31], [32]. Nevertheless, the way in which the complex architecture of a *cis*-regulatory systems (CRS), (binding sites (BSs), transcription factors (TFs), cooperativity mechanisms, DNA-loops) orchestrates the required response is still unknown. Moreover, the evolutionary criteria operating over the latter must also be considered.

In order to achieve a clearer understanding of the principles guiding the design of complex CRS, we previously studied a stochastic model for a single TF capable of binding cooperatively to three regulatory BSs [33], [34]. In these papers, we described two different cooperative binding mechanisms (CBM): the recruitment mechanism, which increases the ability for new TF recruitment, and the stabilisation mechanism, which increases the stability of the TF-DNA bond. We also reported that, at single-gene level, the sensitivity of the output response can be due to multiple regulatory BSs for TFs that act cooperatively [33]. Moreover, we showed that cooperative interactions between TFs increase the intrinsic fluctuations associated with transcription in a mechanism dependent manner [33], [34]. In such papers our study was limited to CRS with three BSs, where each TF interacts with all the TFs which are bound to DNA.

If the design principles of regulatory systems are subject to criteria that maximise sensitivity and minimise noise, our previous results suggested that there could be a trade-off between the number of BSs and the intensity of the cooperative interaction. Thus, the primary purpose of this paper was to explore the existence of this trade-off and, for this, it was necessary to generalise our previous model [33] to consider a variable number of BSs, where each TF could interact with one, two, or more TFs bound to DNA. To study this complex model we developed a small-noise approximation (SNA) framework, introduced in [35], which enables the analysis of arbitrarily complex CRS acting in a small-noise regime.

Our results showed that an increase in the number of BSs can either decrease or increase expression noise depending on the cooperativity intensity and the number of effective TF interactions. Furthermore, we found a scenario where there is an optimal trade-off between cooperativity intensity (a factor that increases noise) and the number of BSs (a factor that decreases noise). The significance of this finding is at least two-fold: from an evolutionary point of view, it represents an alternative functionality criterion mechanism based on noise minimisation, and it also contributes a design principle for synthetic biology projects.

## Methods

### Modeling *cis*-regulatory systems

**Figure 1:**
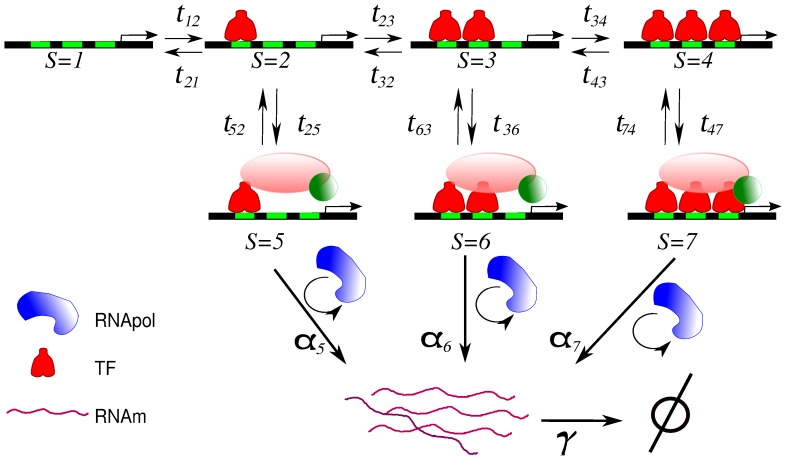
Sketch of the *cis*-regulatory system. For simplicity, we consider a diagram of the promoter region that includes only three regulatory BSs (green boxes) where TF proteins can bind (red molecules). The states on the top layer 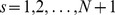 represent states with zero, one, and 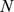 BSs occupied by TFs, respectively. After one or more TFs bind to the regulatory BSs, the regulatory system is able to initiate transcriptional complex formation, where all the components required for transcription (pink and green molecules) are assembled on the CRS. States 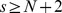 in the middle layer are those capable of recruiting RNApol (blue protein) to synthesise mRNA. The synthesis rate (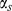) depends on the number of bound TFs. The regulatory system can make transitions from state 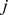 to state 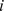 with rate 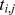. The elements of the transition matrix 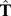 are 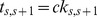 and 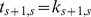 for 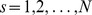, where 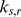 are the kinetic rates.

In order to analyse the effects of tandem BS architecture on transcriptional regulation, we generalised the model used in [33]. Thus, the proposed CRS includes 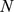 regulatory sites for the same TF. Figure 1 illustrates an example of a CRS that includes three regulatory binding sites, showing the different transitions between states. The states 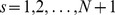 represent, respectively, states with zero, one, and 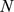 sites occupied by TFs. The states 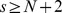 correspond to the transcriptional complex formation, where all components required for transcription are assembled on the CRS. For simplicity, we consider that TFs do not bind or unbind after the formation of the transcriptional complex. Once the core transcription apparatus is formed, the synthesis of one mRNA copy begins. TFs can bind to regulatory sites with a probability which is proportional to the TF concentration 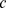 following the law of mass action for elementary reactions. TF unbinding events depend only on the kinetic constants. Thus, the elements of the transition matrix 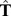 are 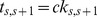 and 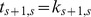 for 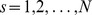, where 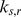 are the kinetic rates.

**Figure 2:**
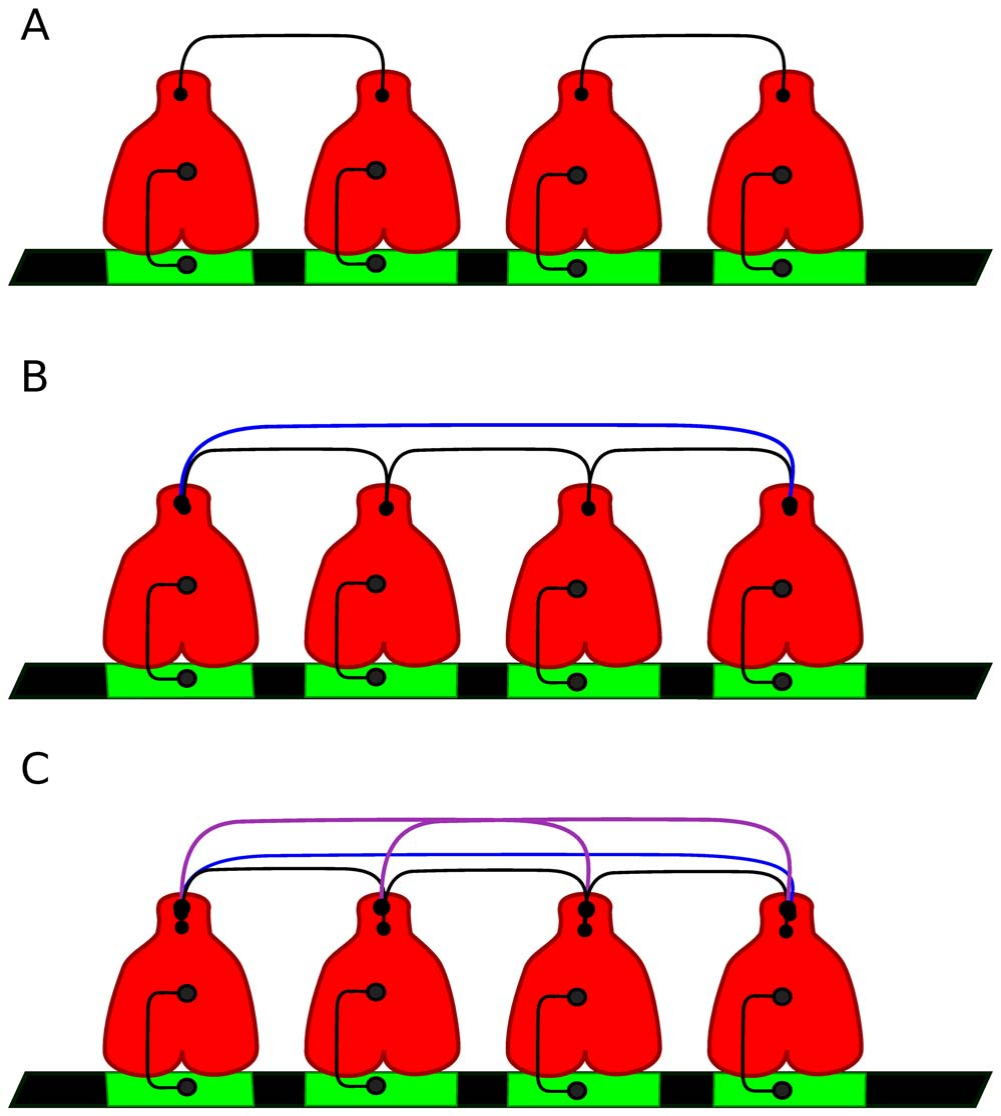
Different number of cooperative interactions. The schematic shows a CRS with four BSs where a bound TF can interact with: (A) only one TF, case (i), (B) two TFs (ii), (C) three other TFs, case (iii).

If we consider cooperative interactions between TFs, kinetic rates are not independent because previous binding alters the actual binding or unbinding process. Known relationships between the system's kinetics and thermodynamic properties (principle of detailed balance) allow us to write the kinetic rates, 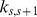 and 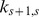 with 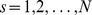, in terms of three parameters [33]: the binding rate 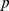, the unbinding rate 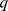, and 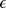 which represents the cooperativity intensity, i.e., (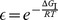, where 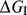 is the free energy of the interaction. Beyond this simplification, this relationship also allows the identification of two CBMs [33]. The first, the recruitment mechanism, occurs when the presence of already bound TFs alter DNA affinities increasing binding rates 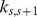, which generates a greater ability for new TF recruitment for DNA binding (sketched in Fig. S1A). The second CBM, the stabilisation mechanism, acts when TF interaction diminishes the unbinding rate 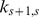, increasing the stability of the TF-DNA interaction (sketched in Fig. S1B). Thus, following [33], we can write



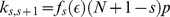


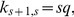
(1)


for the first mechanism, while for the second mechanism we have 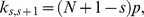


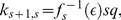
(2)


where 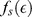 establishes how the cooperative interaction of the state 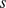 affects the kinetic rates of the new binding or unbinding processes. In previous work we considered the special case of a CRS with three sites, where each TF interacted with all TFs already bound to DNA, and all interactions had the same 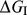 [33], [34]. In this case, 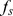 can be written as 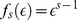 for all 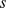. However, due to the spatial distribution of the BSs along the regulatory region, scenarios with a lower number of TF interactions can occur. Even though the CRS model does not include any spatial details, we can emulate several alternative CRS configurations by considering different 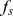:

1. each TF interacts with only one bound TF, that is 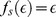 for 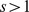;
2. each TF interacts with two bound TFs, that is 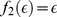 and 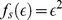 for 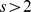;
3. each TF interacts with three bound TFs, that is 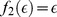, 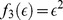 and 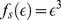 for 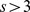.
4. each TF interacts with all bound TFs, that is 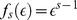 for all 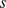. Of course, 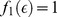 for all cases, since there is no bound TF to interact in the state 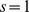. Figure 2 illustrates three regulatory systems, with the same occupancy level, where the number of TF interactions increases from one to three.

### Small-noise approximation

As other authors [36], [35], [33], we use the master equation approach to derive the response of a cell population to an inductive signal. The state of our system will be specified by two stochastic variables: the chemical state of the CRS 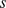, and the number of transcripts 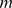, where 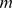 is a positive integer and 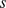 is either 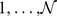. The probability to find the system in the state 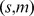, at any time 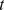, can be written as a vector 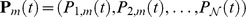. The time evolution for this probability is governed by the following master equation: 
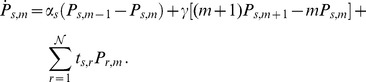
(3)


where 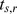 is the transition probability per time unit from state 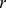 to state 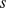. The first two terms correspond to the production and degradation of mRNA, respectively. The model assumes that mRNA is synthesised at rate 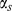 which depends on the state 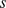, whereas it is degraded linearly with rate 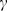. The last term on the right hand side of the equation (3) describes CRS dynamics.

An exact analytical description has been obtained previously for a steady state with 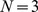 [33], but that is not possible for 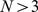 without incorporating some approximations. In this sense, following Kepler and Elston [35], we apply two different approximations to Eq. (3): (i) when the number of transcripts is large, a diffusion approximation is used; (ii) when the CRS transition rates are large compared to the rate of production and degradation of transcripts, an SNA is possible. We now define an appropriately scaled continuous and dimensionless variable 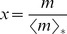, where 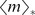 is the mean number of transcripts in the steady state. This allows us to introduce the transformation 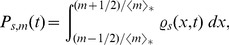
(4)


which defines the probability density function 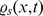. Consequently, Eq. (3) is transformed into an evolution equation for 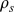. The use of a second order diffusion approximation and then of a first order SNA, leads to an equation for marginal density 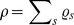, 
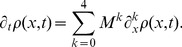
(5)


Neglecting terms of higher order than two on 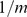 and further algebraic steps, allows us to find expressions for the coefficients 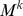 as follows: 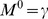, 
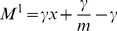
 and 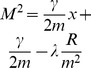, while 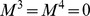 where, to simplify notation, we dropped the angle brackets and the star to denote the mean value of 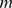 in its steady state, 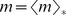. The factor 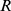 depends on the kinetic rates, 
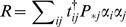
. (see Text S1 for a detailed derivation). 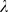 keeps track of the SNA expansion order. Notice that noise due to CRS dynamics, through factor 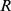, influences the term associated with diffusion in the Fokker-Planck equation, while the deterministic limit is restored when 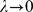. Thus, the master equation can be cast into a Fokker-Planck equation for marginal density 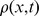


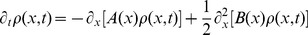
(6)


with 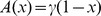 and 
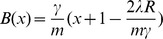
. An important advantage of this formulation is that the associated Fokker-Planck equation has a closed expression for steady state density in terms of a simple quadrature 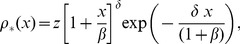
(7)


with 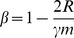, 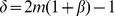 and 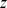 is a normalisation constant; 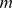 is indicating the average number of transcripts in the steady state. Notice that expression (7) is 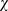-squared and similar to the Gaussian form in the SNA region of validity. The expression (7) is general, meaning that it is valid for any CRS, whatever the dynamics and number of BSs. As solution (7) is an approximated one, an estimation of the SNA accuracy is necessary. For this, we define 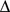 as the ratio between 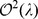 and 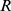 (which is proportional to 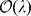). Consequently, our approximation predictions are accurate for small 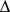 (See Text S1 for details).

## Results

In this section we validated our SNA and analysed fluctuation behaviour and sensitivity as a function of the number of binding sites and 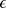 within SNA validity limits.

**Figure 3:**
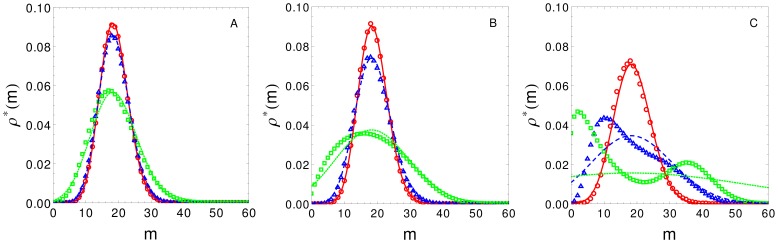
Transcript distributions. Comparison between exact (symbols, obtained by stochastic simulations) and analytical distributions (curves, obtained by the SNA) for three different CRS kinetic rates (red, blue and green correspond to quick, medium and slow CRS dynamics, respectively). Panels from left to right show non-cooperative, recruitment and stabilisation CBMs, respectively. In the last two cases 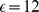. The comparison shows very good correspondence when CRS state transitions are fast in relation to production and degradation rates. This figure shows that the SNA fails to correctly predict the distribution for slow kinetics when there is TF cooperative binding, even though this is more apparent for the stabilisation CBM.

In order to validate the solutions obtained with the proposed approximation, we compared the transcript number analytical distributions predicted by Eq. (4), with the corresponding histograms obtained by stochastic simulation using the Gillespie method [37]. Figure 3 shows the distribution which was obtained for a CRS with three binding sites in which each TF interacts with all TFs already bound to DNA (case iv). Green color corresponds to the parameter values listed in Table 1. To highlight the effect of CRS kinetics on the approximation, we also show the distributions that were obtained with different kinetic rates, by multiplying the elements of matrix 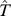 by factor 10 (blue color), and by factor 100 (red color). Fig. 3A corresponds to the distribution obtained for a non-cooperative binding case (i.e. 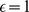), while Fig. 3B and Fig. 3C correspond to recruitment and stabilisation CBMs, respectively, (with 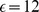). As expected, our results showed excellent correspondence for fast transitions between CRS states in relation to production and degradation rates. When cooperative binding was included in the model (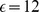), approximation accuracy decreased. Moreover, comparison of Fig. 3B and Fig. 3C shows that the approximation for recruitment CBM is more accurate than for stabilisation CBM. This occurs because stabilisation CBM decreases unbinding rates (i.e., slows down CRS kinetics), as can be observed explicitly in Eq. (1) and Eq. (2). Furthermore, SNA accuracy increases for lower interaction intensity and for a lower number of interactions (data not shown). The comparison between SNA predictions and the corresponding simulation results in Table 2 shows that, when 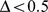, mean and standard deviation estimations for each distribution are reliable.

**Table 1:** Parameter values.

Name	Symbol	Value
TF binding	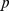	0.25
TF unbinding	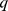	0.75
Coop. intensity	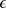	variable
TC assembly 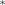	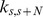	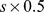
TC disassembly 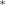	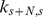	0.5
Transcript prod.	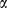	1.5
Transcript degrad.	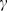	0.03
Number of BS	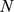	variable

kinetics rates 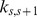 and 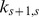, with 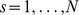, were obtained in each case using the corresponding Eqs. (1) or (2), with the above values of 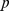, 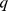 and 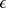. The time unit is min and the concentration is an arbitrary unit. 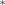 with 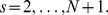

**Table 2:** SNA performance.

	mean value		standard deviation		
plot	simulation	SNA	simulation	SNA	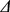
Fig.3A green	18.75	18.75	7.01	6.94	0.134
Fig.3A blue	18.73	18.75	4.68	4.67	0.013
Fig.3A red	18.77	18.75	4.37	4.37	0.001
Fig.3B green	18.75	18.75	10.18	10.02	0.581
Fig.3B blue	18.74	18.75	5.40	5.38	0.058
Fig.3B red	18.77	18.75	4.45	4.43	0.006
Fig.3C green	18.78	18.75	25.77	14.87	12.558
Fig.3C blue	18.71	18.75	10.97	10.04	1.256
Fig.3C red	18.77	18.75	5.65	5.57	0.125

Comparison of the transcript number mean values, and its associated standard deviation, obtained by Gillespie simulations and predicted by SNA for the plots depicted in Figure 3. The last column depict the estimator for SNA accuracy 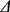.

In this context, we applied SNA to study the performance of a complex CRS using the parameter values listed in Table 1. In particular, we were interested in quantifying the phenotypic noise (spread of expression levels within the cell population) of an activator switch in terms of the CRS architecture (i.e., as a function of the number of BSs and 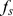), and the intensity of the cooperativity 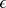 involved in the CRS. In this sense, to characterise noise expression we computed the fluctuation/signal ratio (which is known as noise) defined by 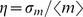 [38], at an activator concentration 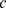 equal to the 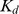 of the dose-response curves. Given the SNA region of validity, hereafter both 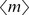 and 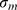 were obtained from SNA distributions with 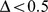.

**Figure 4:**
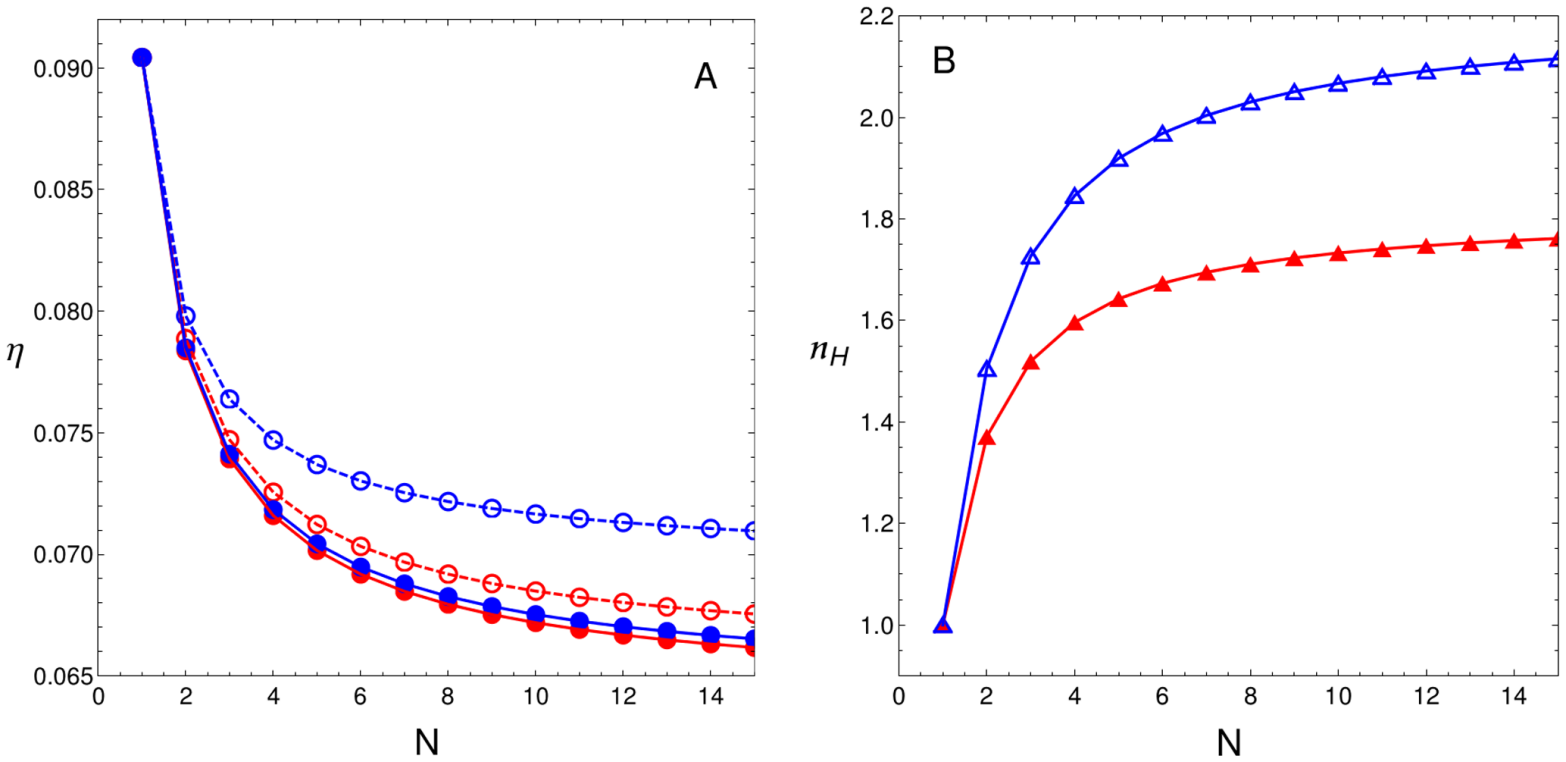
Case (i), TFs interact with only one other TF. When 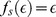 for 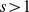, noise 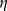 and steepness 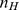 behaviour is monotonic with respect to the number of BSs 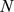. (A) 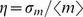 as a function of 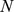 for both CBMs (filled and open circles indicate recruitment and stabilisation CBMs, respectively) with 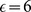 (red circles) and 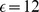 (blue circles). (B) 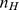 as a function of 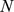 for 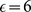 (red triangles) and 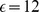 (blue triangles). Curves correspond to cubic spline interpolations.

**Figure 5:**
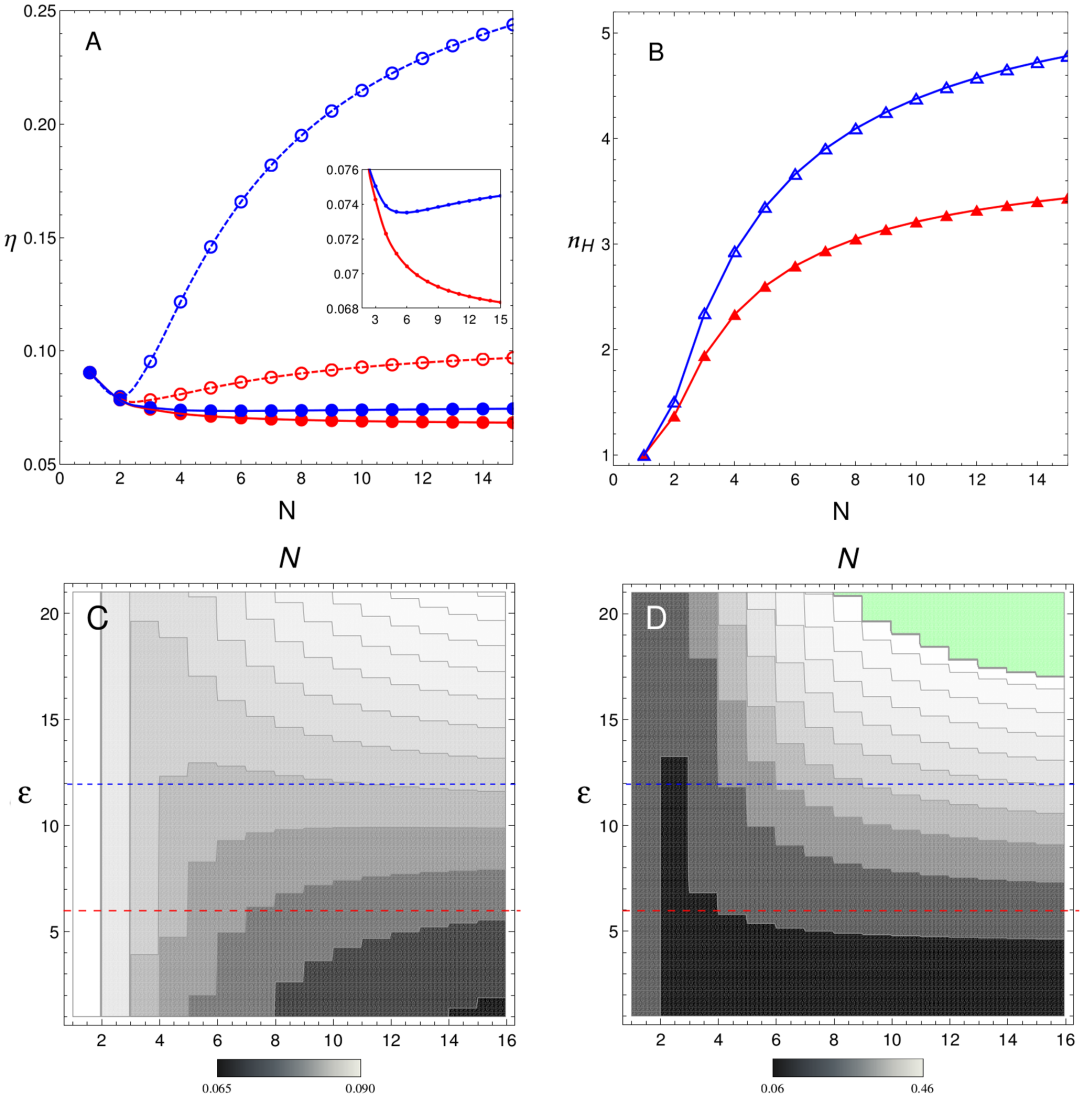
Case (ii), each TF can interact with up to two other TFs. When 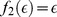 and 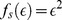 for 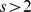, noise 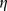 shows a more complex behaviour which depends on the acting CBM and the intensity of cooperativity. (A) 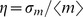 as a function of the number of sites 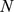 for both CBMs (filled and open circles indicate recruitment and stabilisation CBMs, respectively) with 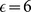 (red circles) and 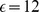 (blue circles). The inset rescales the recruitment case for both 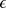-values. (B) 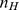 as a function of 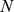 for 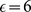 (red triangles) and 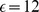 (blue triangles). Curves correspond to cubic spline interpolations. Lower panels correspond to density plots of 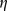 as function of 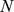 and 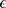 for recruitment CBM (C) and stabilisation CBM (D). Dotted lines indicate the values of 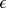 used in panels A and B. For recruitment CBM, the density plot clearly shows the existence of a valley in 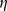 around 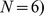 for 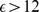 while, for stabilisation CBM, the valley in 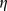 is apparent for lower values of 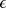 around 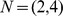. The green area in panel D denotes the region where 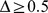 and SNA predictions are less accurate.

Fig. 4 depicts two important features of the regulatory system response, as a function of 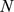: the noise 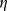 (A) and the Hill coefficient 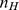 of the mean response (B). These features are presented for both CBMs: recruitment (filled circles) and stabilisation (open circles). Since the Hill coefficient is CBM-independent, curves in panel B are superimposed. Fig. 4 illustrates case (i), where each TF interacts with only one bound TF. This figure shows how 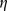 decays monotonically (Fig. 4A) while the steepness of the associated response (Fig. 4B) increases with the number of BSs 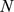 for all curves. The red and blue curves correspond to 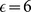 and 12, respectively. Similar behaviour can be found for other 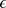 values. This is illustrated by the density plot (on a plane) shown in Figure S2 for both CBMs 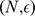. This behaviour suggests that, when TFs interact with only one other TF, additional BSs on the CRS improve the signal/noise ratio and the sensitivity of the switch response (the latter is characterised by the Hill coefficient). However, when CRS architecture admits more than one interaction between TFs, noise, as a function of 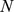, shows a complex behaviour. Fig. 5A depicts noise for case (ii), in which each TF interacts with two bound TFs. In this case, 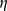 behaviour depends on which CBM is acting and on the intensity of this cooperativity, 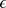. Red and blue curves correspond to 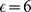 and 12, respectively, while the filled and open circles correspond to recruitment and stabilisation CBMs, respectively. For the cases illustrated in Fig. 5A, noise 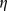 shows a minimum around 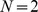 when stabilisation CBM is acting, and 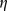 increases with 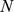 for 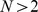. When recruitment CBM is acting, 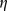 can show a minimum in an 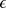 dependent manner, or not, as can be observed in Fig. 5A inset. At 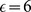 (red curve) 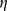 decays monotonically with 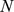 and there is no valley. However, for a higher intensity of cooperativity (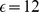), 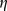 has a minimum around 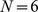. On the other hand, similarly to Fig. 4B, Hill coefficient associated to the steepness of the mean response, increases with the number of BSs 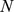, as is shown in Fig. 5B. However, the steepness for case (ii) is always higher than for case (i). This means that Hill coefficients 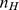 are determined not only by the number of BSs in the regulatory system and the interaction energy between TFs, but also by the number of TF interactions which are admitted by the CRS. Noise behaviour in an 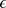 dependent manner can be better illustrated by density plots on a plane 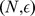, as those depicted in the bottom panels of Fig. 5. Fig. 5C and Fig. 5D show 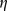 in grey scale maps as a function of 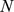 and 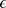 for both CBMs. For recruitment CBM (Fig. 5C), a valley appears when 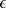 is greater than 10 and the position of the minimum changes with 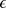. For example, for 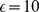 there is a valley around 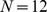, whereas for 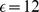 (blue dotted line) the valley is around 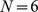, and for 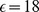 the minimum position shifts to 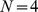. For stabilisation CBM (Fig. 5D), the minimum is more apparent at a lower intensity of cooperativity. For example there is a minimum at 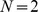 for 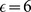 (red dotted line), although this valley also exists at smaller 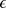 (data not shown).

**Figure 6:**
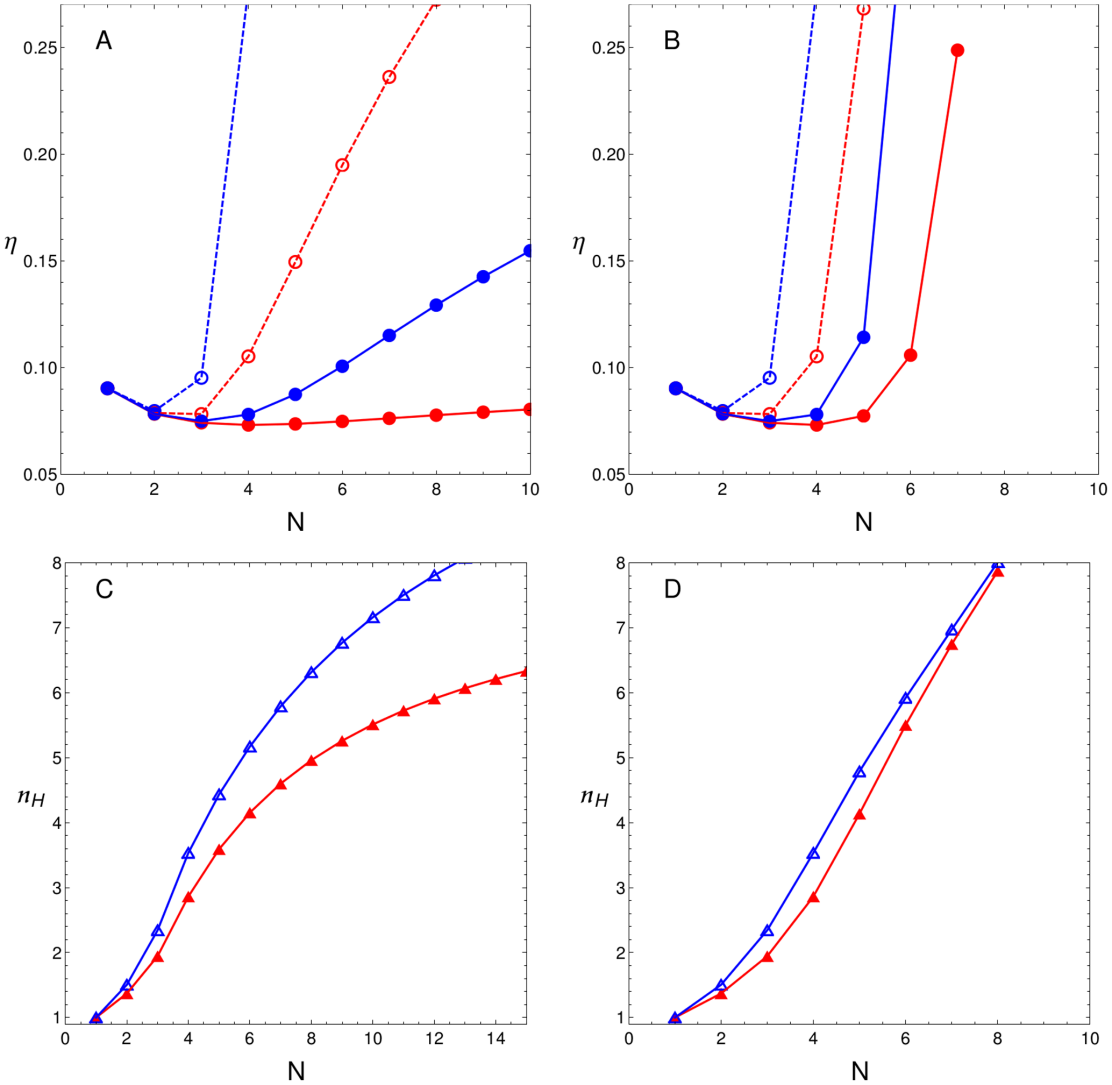
Cases with many interactions. Top panels: 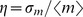 as a function of the number of sites 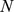 for both CBMs (filled and open circles indicate recruitment and stabilisation CBMs, respectively) with 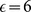 (red circles) and 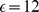 (blue circles). Bottom panels: 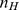 as a function of 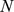 for 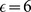 (red triangles) and 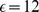 (blue triangles). Panels A and C show the case in which each TF can interact with up to three other TFs, i.e., case (iii). Panels B and D show the case where each TF interacts with all available TFs (case (iv)) where 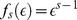 for all 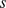. Curves correspond to cubic spline interpolations.

Fig. 6 depicts noise 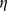 (A) and Hill coefficients 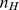 (B) as a function of 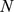 for case (iii), where each TF interacts with three bound TFs. Figs. 6B and 6D depict case (iv), where each TF interacts with all bound TFs. The red and blue curves correspond to 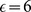 and 12, respectively, while the open and filled circles correspond to recruitment and stabilisation CBMs, respectively. Both CBMs show a minimum in the fluctuation/signal ratio for 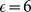. However, the number of BSs necessary for an optimal performance differ between CBMs: the minimum for recruitment CBM is around 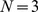 or 4, depending on 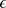, while for stabilisation CBM the minimum is around 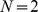. Thus, for many interactions, as cases (iii) and (iv), the signal/noise ratio is maximised by an architecture with two or three binding sites. Minima in 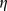 can also be found for faster CRS with weaker cooperativity (data not shown). Moreover, it can also be observed that the sensitivity of the switch response (characterised by the Hill coefficient) increases faster with 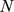 than in previous cases. In particular, for case (iv) (Fig. 6D), 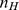 approximation by 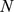 is accurate only for a high interaction energy and 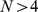. Finally, Figures 4, 5 and 6 suggest that noise and steepness increase with the number of TF interactions which are admitted by the CRS.

## Discussion

Regulation of gene expression is a topic of central importance in biology. Given our increasing ability to monitor gene expression levels and model its regulation, many key issues have been unraveled in the past years. An example of this is the discrete nature of the transcriptional process which impacts on the noise expression phenomenon and has promoted the extended use of stochastic modeling to study the origin of noise expression and its propagation. However, many other issues require new models and methodological approaches to be elucidated. For example, an important aspect which impacts synthetic biology, is to understand how the CRS architecture (i.e., the biological components of the regulatory system and its organisation) determines the regulatory function features and the associated fluctuations. Most of the previous studies which analysed gene product fluctuations due to transitions between CRS states, used models which considered a small number of states for the CRS. This is mainly because theoretical approaches become intractable for complex CRS [34], [39], but the use of simple models can limit our understanding of gene regulatory phenomena involving complex CRS. However, modeling of complex CRS is becoming more frequent in specialised literature [39], [40], [41].

This paper had mainly two aims: the first was methodological and consisted in presenting an approximation of the master equation to deal with stochastic models for arbitrarily complex CRS in an analytical fashion. In this respect, we developed the SNA for a generic CRS which can include many states. Thus, we derived an explicit form for the Fokker-Plank equation from a microscopic description which can be applied to any CRS. This approximation was validated against the distribution of mRNA levels generated by a Gillespie simulation and showed very good correspondence in the unimodal regime. Other approaches have recently been developed which are capable of dealing with complex CRS, among these, the stochastic spectral analysis [42] and the effective rate equation approach developed by Grima [43]. These approaches could deal with bimodal distributions more accurately than SNA.

The second and most important aim, was to study the impact of tandem regulatory BSs on CRS response to TF activators, using the above mentioned approximation. Namely, how noise and sensitivity depend on the number of regulatory BSs. At this point it is important to distinguish regulatory BSs from decoy BSs that competitively bind TFs. Decoy sites protect these proteins from degradation and thereby indirectly influence the expression of target promoters. The role of decoy BSs was studied recently by [44], [45], who showed that protective decoys can buffer noise by reducing correlations between TFs. Hitherto, the role of the number of regulatory BSs on the regulation of expression level and its associated fluctuation, have only been addressed by a few studies. In one of these studies, experimental evidence from genetically modified mammalian cells showed that increases in the number of regulatory BSs lead to higher noise (Fig. 3B of [46]). However, the contrary effect was reported in Drosophila, where an increasing number of Bcd BSs decreased noise (Fig. 6 of [41]). A more recent study which analysed the expression level and noise associated with several native targets of the transcription factor Zap1 [32], suggested that the relationship between expression level and noise is a feature that characterises the CRS and is determined by CRS architecture. However, in all these studies the precise mechanisms by which noise is controlled remained unidentified.

In this context, we previously reported that noise expression behaviour depends on the acting CBM and on cooperativity intensity [33]. Our present results expand upon this analysis showing that CRS performance is a consequence of the interplay between diverse factors: CBMs, the number of regulatory BSs, and the number and intensity of TF interactions. Figures 4 and 5 show that increasing the number of regulatory BSs can lead to noise reduction in a low interaction scenario. We also show that the type of acting CBM and the number of interactions play a critical role in determining how noise 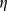 depends on the number of regulatory BSs. Finally, we identified a scenario where noise presents a minimum on a plane defined by 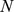 and 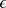, where the size of the valley and its position depend on the number of interactions and the CBM. Moreover, the position of the valley was found around intervals [2], [6] for 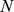, and [3], [20] for 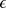 (corresponding to 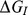 ranging from 0.65–1.8 kcal/mol). Since the mentioned values for 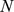 and 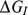 correspond to those found in the spectrum of real biological systems, our results suggest that evolutionary processes are capable of adjusting these parameters to optimise noise in single-gene switches.

## Supporting Information

Figure S1
**Cooperative binding mechanisms.** The effect of cooperative binding on binding and unbinding. (A) In the recruitment mechanism, TFs already bound to the DNA increase the ability for recruiting new TFs. (B) In the stabilisation mechanism, TF interaction diminishes the unbinding rate. 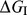 denotes the free energy involved in the cooperative binding; the black link represents a chemical interaction.(TIF)

Figure S2
**Density plots for case (i).** 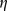 as function of 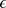 and 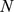: for recruitment (panel A) and stabilisation (panel B) CBMs. Dotted lines indicate the values of 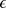 used in Figs. 4A and 4B. The density plots clearly show that there is no valley in 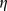 for the explored parameters.(TIF)

Text S1 **Supporting Information.**
(PDF)
