# Supplementary figures and images for: Noise Minimisation in Gene Expression Switches
Source: PLoS One. 2013 Dec 23;8(12):e84020. doi: 10.1371/journal.pone.0084020 (PMC3871557; doi:10.1371/journal.pone.0084020)

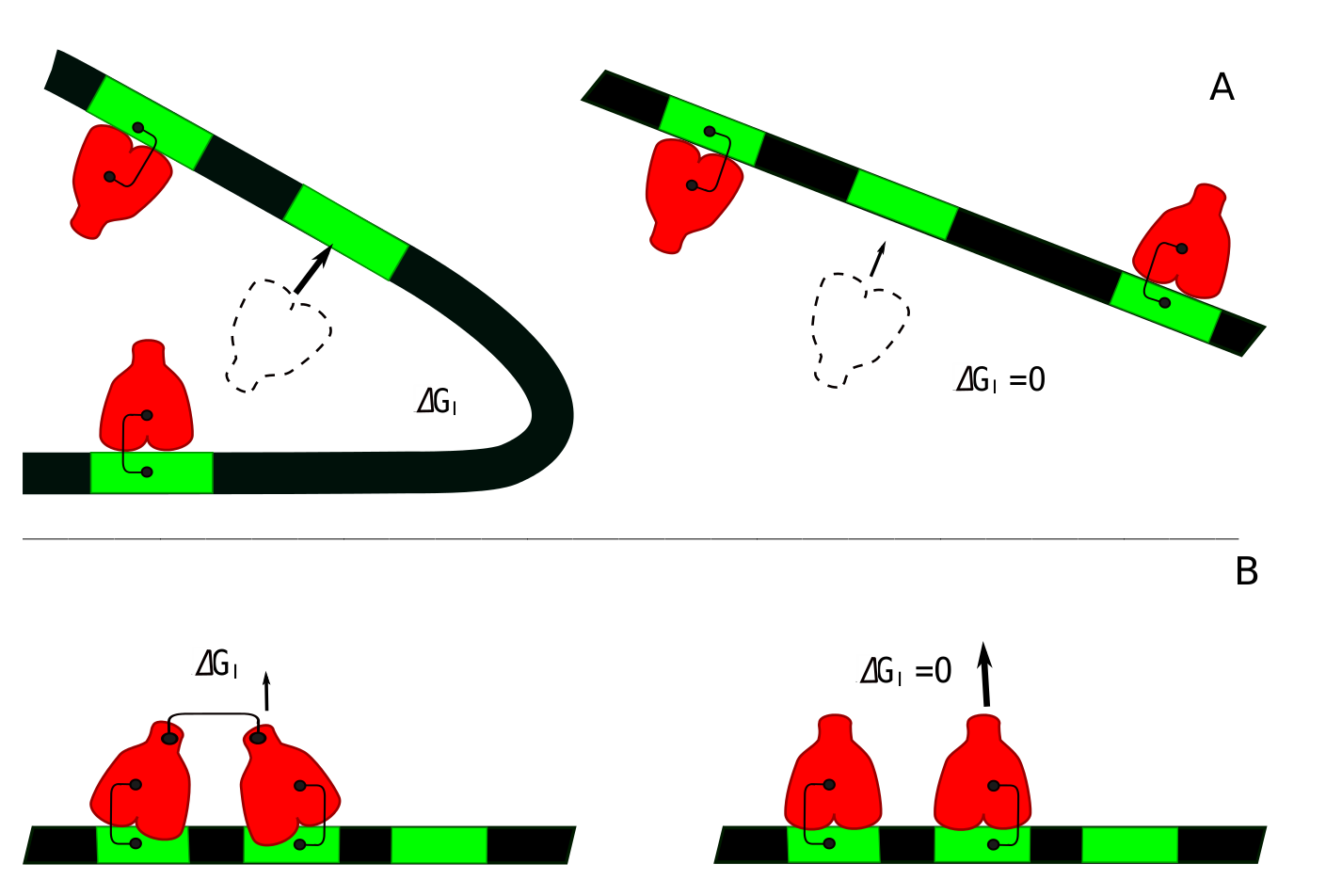

Supplement: Figure S1 — Cooperative binding mechanisms. The effect of cooperative binding on binding and unbinding. (A) In the recruitment mechanism, TFs already bound to the DNA increase the ability for recruiting new TFs. (B) In the stabilisation mechanism, TF interaction diminishes the unbinding rate. denotes the free energy involved in the cooperative binding; the black link represents a chemical interaction. (TIF) [file pone.0084020.s001.tif]

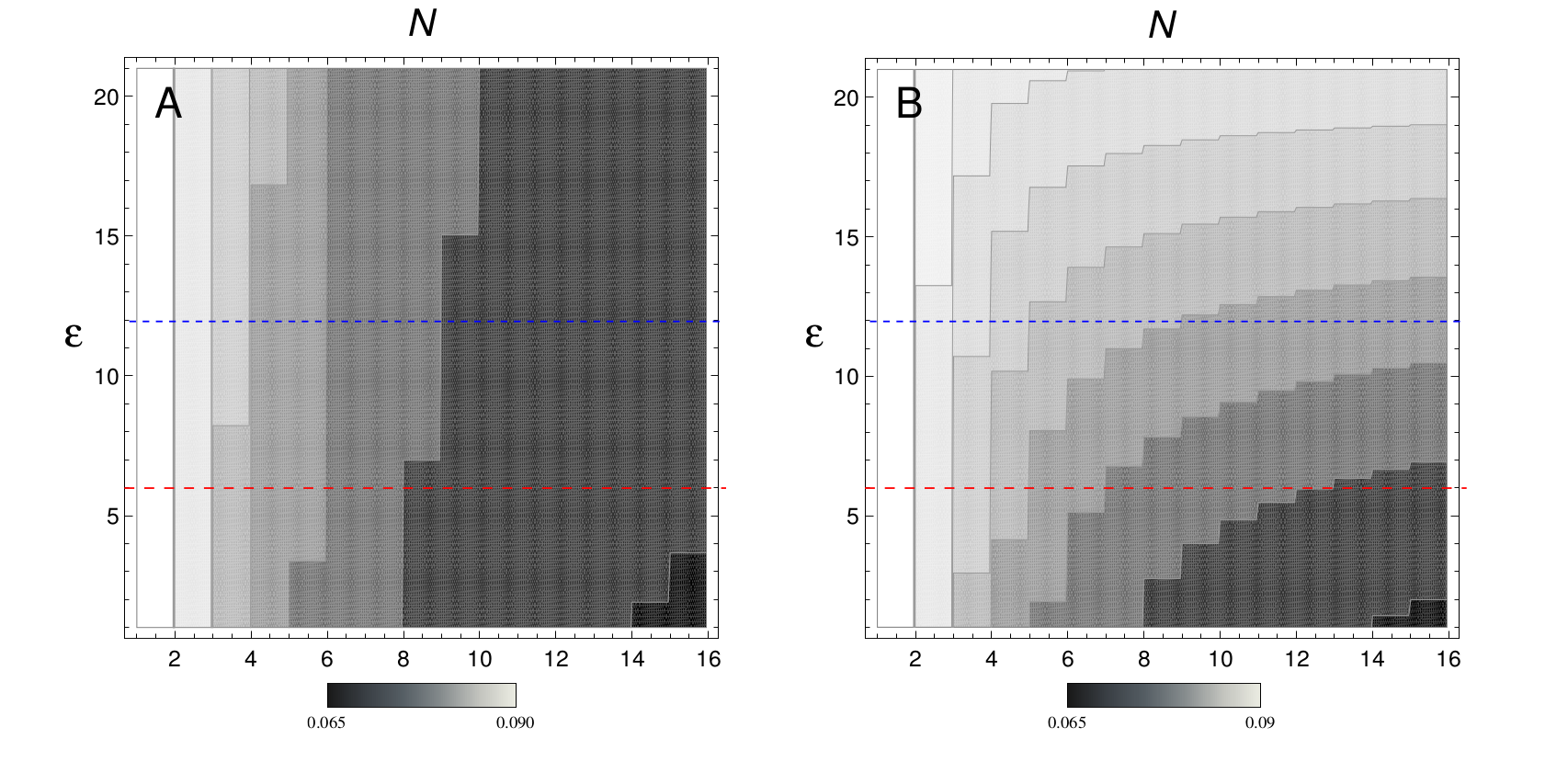

Supplement: Figure S2 — Density plots for case (i). as function of and : for recruitment (panel A) and stabilisation (panel B) CBMs. Dotted lines indicate the values of used in Figs. 4A and 4B. The density plots clearly show that there is no valley in for the explored parameters. (TIF) [file pone.0084020.s002.tif]
