# Supplementary material for: Noise Minimisation in Gene Expression Switches
Source: PLoS One. 2013 Dec 23;8(12):e84020. doi: 10.1371/journal.pone.0084020 (PMC3871557; doi:10.1371/journal.pone.0084020)
Supplement: Text S1 — Supporting Information. (PDF) [file pone.0084020.s003.pdf]

Supporting Information for *Noise Minimization in Gene Expression Switches*

Diana Monteoliva, Christina B. McCarthy and Luis Diambra

Text S1

The state of our system is determined by the chemical state of the CRS, denoted by s and the number of transcripts m. The probability of finding the system in the state (s, m) at a time t, is $\mathbf{P}_m(t) = (P_{1,m}(t), P_{2,m}(t), \ldots, P_{\mathcal{N}}(t))$. The temporal evolution of $\mathbf{P}_m(t)$ is governed by the master equation:

$$\dot{P}_{s,m} = \alpha_s (P_{s,m-1} - P_{s,m}) + \gamma [(m+1)P_{s,m+1} - mP_{s,m}] + \sum_{r=1}^{\mathcal{N}} t_{s,r} P_{r,m} \,. \quad (1)$$

where $t_{s,r}$ is the transition probability per time unit from state r to state s, while the first two terms correspond to the production and degradation of mRNA, respectively. We will apply two different approximations to Eq. (1): the diffusion approximation and the small-noise approximation (SNA).

Diffusion approximation: We now define an appropriately scaled continuous variable $x = \frac{m}{\overline{m}_*}$, where $\overline{m}_*$ is the average number of transcripts in the steady-state. As defined here, x is dimensionless, though we shall often refer to it as a concentration. We define the probability density function $\varrho_s(x, t)$ as

$$P_{s,m}(t) = \int_{(m-1/2)/\overline{m}_*}^{(m+1/2)/\overline{m}_*} dx \; \varrho_s(x, t) \quad (2)$$

Changing variables and making use of the shift operator $e^{u\partial_x}$, which translates the function f from x to $x + u$, on Eq. (1), we obtain the evolution equations for ϱ_s,

$$\partial_t \varrho_s(x) = \alpha_s \left(e^{-\frac{1}{m}\partial_x} - 1\right) \varrho_s(x) + m\gamma \left(e^{\frac{1}{m}\partial_x} - 1\right) (x\varrho_s(x)) \quad (3)$$

where, to simplify notation, we have dropped the bars and stars to denote the mean value of m in its steady state, $m = \overline{m}_*$. When the gene product

level m is large, we can expand the shift operator as a Taylor series neglecting terms of order three on $\frac{1}{m}$ and higher to obtain the second order diffusion approximation

$$\partial_t \boldsymbol{\varrho} = \mathbf{L}(x)\boldsymbol{\varrho} + \hat{\mathbf{T}}\boldsymbol{\varrho}\,, \tag{4}$$

where $\boldsymbol{\varrho}$ is the $\mathcal{N}$-dimensional vector of components ϱ_s, $\hat{\mathbf{T}}$ is the matrix operator representing the CRS dynamics and $\mathbf{L}(\mathbf{x})$ is a diagonal matrix operator with elements

$$L_{s,s} = -\partial_x g_s(x) + \frac{1}{2}\partial_x^2 h_s(x) \tag{5}$$

with $\mathbf{g}$ and $\mathbf{h}$ $\mathcal{N}$-dimensional vectors whose components are

$$g_s = \frac{\alpha_s}{m} - \gamma x \quad \text{and} \quad h_s = \frac{1}{m}\left(\frac{\alpha_s}{m} + \gamma x\right)\,. \tag{6}$$

Small-noise approximation: Often CRS state transitions occur at a different time-scale than the production and degradation rates for transcripts. In order to make this scaling explicit we substitute $\hat{\mathbf{T}}$ by $\hat{\mathbf{T}}/\lambda$. The parameter λ keeps track of the expansion order of the SNA and allows scaling all CRS kinetics with respect to the production and degradation kinetics. Thus we rewrite Eq. (11) as:

$$\partial_t \boldsymbol{\varrho} = \mathbf{L}(x)\boldsymbol{\varrho} + \frac{1}{\lambda}\hat{\mathbf{T}}\boldsymbol{\varrho}\,, \tag{7}$$

Following [S1], we assume that the probabilities for the operator states reach their steady-state values before x changes appreciably. We can obtain an equation for the marginal density $\rho = \sum_s \varrho_s$, by expanding it as a power series in λ

$$\partial_t \rho = \mathbf{1}^T \mathbf{L}\mathbf{P}_* \rho - \lambda \mathbf{1}^T \mathbf{L}\hat{\mathbf{T}}^\dagger \mathbf{L}\mathbf{P}_* \rho + \mathcal{O}^2(\lambda), \tag{8}$$

where $\mathbf{P}_*$ is the right eigenvector of the matrix operator $\hat{\mathbf{T}}$ associated to the eigenvalue zero (i.e. $\hat{\mathbf{T}}\mathbf{P}_* = \mathbf{0}$), and

$\hat{\mathbf{T}}^\dagger$ is the pseudoinverse of $\hat{\mathbf{T}}$. The first term on the right hand side of Eq. (8) corresponds to the deterministic limit with $\lambda \to 0$, while the second term represents the influence of noise on the dynamics. Making use of expressions (5) and (6), after some long but straightforward calculations Eq. (8) can be written as

$$\partial_t \rho(x,t) = \sum_{k=0}^{4} M^k \partial_x^k \rho(x,t) \tag{9}$$

with

$$
\begin{aligned}
M^0 &= \gamma - \lambda\gamma^2 R_1 \\
M^1 &= (\gamma - 3\lambda\gamma^2 R_1)x + \frac{\gamma}{m} - \gamma + \lambda(2R_2 + R_3) \\
M^2 &= -\lambda\gamma^2 R_1 x^2 + (\frac{\gamma}{2m} + \lambda(\frac{\gamma}{m}(R_2 + R_3) - 9\frac{\gamma^2}{2m}R_1))x + \\
&\quad + \frac{\gamma}{2m} - \lambda[\frac{R_4}{m^2} + \frac{\gamma R_3}{2m^2} - \frac{3\gamma^2 R_1}{2m^2}] \qquad (10) \\
M^3 &= -\lambda\frac{\gamma^2}{m}R_1 x^2 - \frac{3}{2}\lambda\frac{\gamma^2}{m^2}R_1 x + \lambda[\frac{R_4}{m^3} - \frac{\gamma}{2m^3}(2R_2 + R_3)] \\
M^4 &= -\lambda\frac{\gamma}{4m^4}R_1 x^2 - \lambda\frac{\gamma}{4m^3}(R_2 + R_3)x - \lambda\frac{1}{4m^4}R_4
\end{aligned}
$$

where the R_k are defined as $R_1 = \sum_{ij} t^\dagger_{ij} P_{*j}$, $R_2 = \sum_{ij} t^\dagger_{ij} P_{*j}\alpha_i$, $R_3 = \sum_{ij} t^\dagger_{ij} P_{*j}\alpha_j$ and $R_4 = \sum_{ij} t^\dagger_{ij} P_{*j}\alpha_i\alpha_j$, while $t^\dagger_{ij}$ are the matrix elements of the pseudoinverse matrix operator $\hat{\mathbf{T}}^\dagger$. At this point it could be useful to bear some properties of the $\hat{\mathbf{T}}$ pseudoinverse in mind,

$$
\hat{\mathbf{T}}^\dagger \cdot \hat{\mathbf{T}} = \hat{\mathbf{T}} \cdot \hat{\mathbf{T}}^\dagger = \hat{\pi} \quad \hat{\mathbf{T}}^\dagger \hat{\pi} = \hat{\mathbf{T}}^\dagger \qquad \hat{\pi} = \hat{\mathbf{I}} - \mathbf{P}_* \mathbf{1}^T \qquad (11)
$$

and $\hat{\pi}$ is the projection operator which projects out the dynamics of the system which does not lie in the null space of $\hat{\mathbf{T}}$. An explicit repression for $\hat{\mathbf{T}}^\dagger$ is

$$
\hat{\mathbf{T}}^\dagger = \hat{\mathbf{E}}\mathbf{\Lambda}\hat{\mathbf{E}}^{-1} \qquad (12)
$$

where $\hat{\mathbf{E}}$ is the matrix whose columns are the right eigenvectors of $\hat{\mathbf{T}}$ and $\mathbf{\Lambda}$ is the diagonal matrix whose entries are the inverse of the $\hat{\mathbf{T}}$ eigenvalues, except when the entry corresponding to the null eigenvalue is itself zero.

Now we will verify that all parameters R_k, with the exception of R_4, are zero. From the definition of $\mathbf{P}_*$, $\hat{\mathbf{T}}\mathbf{P}_* = \mathbf{0}$, then $\hat{\mathbf{T}}^\dagger\hat{\mathbf{T}}\mathbf{P}_* = (\hat{\mathbf{T}}^\dagger\hat{\mathbf{T}})\mathbf{P}_* = \pi\hat{\mathbf{P}}_* = \mathbf{0}$, where we have made use of relationships (11). Applying the second relationship (11), $\hat{\mathbf{T}}^\dagger\mathbf{P}_* = (\hat{\mathbf{T}}^\dagger\pi)\mathbf{P}_* = \hat{\mathbf{T}}^\dagger(\pi P_*) = \mathbf{0}$. As $R_1 = \sum_{ij} t^\dagger_{ij} P_{*j} = \sum_i (\hat{\mathbf{T}}^\dagger\mathbf{P}_*)_i$, then $R_1 = 0$.

For R_2 in a similar way we have $\sum_{ij} t^\dagger_{ij} P_{*j}\alpha_i = \sum_i (\hat{\mathbf{T}}^\dagger\mathbf{P}_*)_i\alpha_i = 0$. For R_3 we have $\sum_{ij} t^\dagger_{ij} P_{*j}\alpha_j = \sum_j (\hat{\mathbf{T}}^\dagger)_j P_{*j}\alpha_j = \sum_i (\mathbf{1}^T\hat{\mathbf{T}}^\dagger)_j\alpha_j$, as the probability is conserved, $\mathbf{1}^T\hat{\mathbf{T}} = \mathbf{0}$, then we have $R_3 = 0$. But for R_4 we have that $\sum_{ij} t^\dagger_{ij} P_{*j}\alpha_i\alpha_j$ is zero only if $\alpha_i = \alpha_j = \alpha\ \forall i, j$; whenever there is some $i \neq j$ such that $\alpha_i \neq \alpha_j$, then R_4 is not zero. Consequently, the coefficients M_k

can be reduced to a simpler form

$$\begin{aligned} M^0 &= \gamma \\ M^1 &= \gamma x + \frac{\gamma}{m} - \gamma \\ M^2 &= \frac{\gamma}{2m}x + \frac{\gamma}{2m} - \lambda\frac{R_4}{m^2} \\ M^3 &= \lambda\frac{R_4}{m^3} \\ M^4 &= -\lambda\frac{1}{4m^4}R_4. \end{aligned} \tag{13}$$

Since we are only keeping terms up to second order on $(\frac{1}{m})$, M_3 and M_4 vanish. Thus, the influence of noise is seen only in the term with second order derivatives on ϱ, through the R_4 parameter. Hereafter, we will drop the subindex of R_4, to call it simply R. The $\mathcal{O}^2(\lambda)$ terms in the expression for M^k, are proportional to $2\gamma\sum_{i,j,k}\hat{\mathbf{T}}^{\dagger}_{i,j}\hat{\mathbf{T}}^{\dagger}_{j,k}\mathbf{P}_{*k}\alpha_i\alpha_k$. This closed expression allows us to define an analytic computable estimator for the accuracy of the SNA as the ratio $\Delta = 2\gamma\sum_{i,j,k}\hat{\mathbf{T}}^{\dagger}_{i,j}\hat{\mathbf{T}}^{\dagger}_{j,k}\mathbf{P}_{*k}\alpha_i\alpha_k/R$.

Simplifications on M^k allow us to derive a Fokker-Planck equation

$$\partial_t\rho(x,t) = -\partial_x[A(x)\rho(x,t)] + \frac{1}{2}\partial_x^2[B(x)\rho(x,t)] \tag{14}$$

with $A(x) = \gamma(1-x)$ and $B(x) = \frac{\gamma}{m}(x+1-\frac{2\lambda R}{m\gamma})$. We can associate a potential $\phi(x)$ to coefficient $A(x)$

$$A(x) = -\partial_x\phi(x) \quad \text{with} \quad \phi(x) = \frac{\gamma}{2}[(x-1)^2-1] \tag{15}$$

that can be thought of as effective free energy for the system. The steady-state of this system is always represented by a stable minimum of ϕ, which is a quadratic function of x.

The associated Fokker-Planck equation has a closed expression for the steady-state distribution given by

$$\rho_*(x) = \mathcal{Z}\left[1+\frac{x}{\beta}\right]^{\delta}\exp\left(-\frac{\delta\ x}{(1+\beta)}\right), \tag{16}$$

with $\beta = 1-\frac{2R}{\gamma m^*}$, $\delta = 2m^*(1+\beta)-1$ and $\mathcal{Z} = \frac{2^z(\beta m^*)^z e^{-2\beta m^*}\Gamma(1-z)}{\beta(\pi\csc(2\pi z)+z\Gamma(-z)(\Gamma(z)-\Gamma(z,2\beta m^*))}$, where $z = 2(1+\beta)m^*$. Once again * indicates the steady-state. Notice that $R<0$ and $\beta>1$.

The mean value and variance of x can also be explicitly calculated as

$$\bar{x} = \frac{\beta 2^{z-1} e^{-2\beta m^*} (\beta m^*)^z \left(1 + 2e^{2\beta m^*} \mathcal{E}_{-z}(2\beta m^*)\right) \Gamma(1-z)}{\frac{z}{2}(\pi \csc(z) + z\Gamma(-z)(\Gamma(z) - \Gamma(z, 2\beta m^*))} \tag{17}$$

$$\begin{aligned} \sigma_x^2 &= \frac{4^z e^{-4m^*\beta}(m^*\beta)^{2(1+z)}}{(m^*z)^2\Gamma^2(z, 2m^*\beta)} \Big(\big[1 + 2m^* e^{2m^*\beta} \mathcal{E}_{-z}(2\beta m^*)\big]^2 + \\ &+ \frac{2ze^{2m^*\beta}\mathcal{E}_{1-z}(2\beta m^*)}{(z+1)(z+2)} \Big[2m^* e^{2m^*\beta}(1 + \beta + 2m^*)(1 + m^* + m^*\beta) \times \\ &\times \mathcal{E}_{-1-z}(2\beta m^*) - 1 + m^* + 2m^{*2} - m^*\beta + 2m^{*2}\beta\Big]\Big). \end{aligned} \tag{18}$$

Reference

S1. Kepler TB, Elston TC (2001) Stochasticity in transcriptional regulation: origins, consequences, and mathematical representations. Biophysical Journal 81: 3116–3136.
